# *Rhaponticum carthamoides* Transformed Root Extract Has Potent Anticancer Activity in Human Leukemia and Lung Adenocarcinoma Cell Lines

**DOI:** 10.1155/2018/8198652

**Published:** 2018-12-09

**Authors:** Ewa Skała, Ewelina Synowiec, Tomasz Kowalczyk, Tomasz Śliwiński, Przemysław Sitarek

**Affiliations:** ^1^Department of Biology and Pharmaceutical Botany, Medical University of Lodz, Muszynskiego 1, 90-151 Lodz, Poland; ^2^Laboratory of Medical Genetics, Faculty of Biology and Environmental Protection, University of Lodz, Pomorska 141/143, 90-236 Lodz, Poland; ^3^Department of Genetics, Plant Molecular Biology and Biotechnology, University of Lodz, Banacha 12/16, 90-237 Lodz, Poland

## Abstract

*Rhaponticum carthamoides* (Willd.) Iljin. is an endemic plant species, which is important in Siberian medicine. It possesses adaptogenic properties and has been used for treatment of overstrain and weakness after illness, physical weakness, and mental weariness. The roots of this species obtained after *Agrobacterium rhizogenes* transformation are rich in caffeoylquinic acid derivatives known as strong antioxidant compounds. The study makes the first evaluation of the cytotoxic and genotoxic activity of transformed root extract (*Rc* TR extract) in various human cancer cell lines: leukemia cells (K-562 and CCRF-CEM) and lung adenocarcinoma cells (A549). It was found that *Rc* TR extract inhibited the cell viability of all tested cell lines in a concentration-dependent manner, and leukemia cell lines were more sensitive to plant extract than A549 lung cancer cell line. Additionally, the *Rc* TR extract reduced the mitochondrial membrane potential and demonstrated genotoxicity against tested cell lines by increasing mitochondrial DNA lesions in *ND1* and *ND5* genes and causing nuclear DNA damage in *TP53* gene. Our results show that *Rc* TR extract may effectively treat cancer cells by inducing dysfunction of mitochondria. Additionally, the role of mtDNA may be a promising factor in chemotherapy, and it needs further studies.

## 1. Introduction

The plant antioxidant compounds have long been known to have beneficial effects on human health; however, recent studies indicate that they may also cause apoptosis and death of cancer cells [1]. The plants contain various classes of secondary metabolites and may be used in cancer therapy. The advantage of plant compounds is their low toxicity or complete absence, and they reduced side effects and are inexpensive [2]. One plant showing a wide spectrum of biological activity is *Rhaponticum carthamoides*; it has been found to demonstrate antioxidant activity and is capable of inducing apoptosis in glioma cells by disrupting mitochondrial membrane potential and increasing ROS level, altering Bax/Bcl-2 level, activating p53, caspase-3, or caspase-9, inducing DNA damage or PARP cleavage, and increasing the level of phosphorylated H2A.X [3–5].

*Rhaponticum carthamoides* (Willd.) Iljin (Asteraceae) is an endemic plant species whose roots and rhizomes have been used for many years in traditional Siberian medicine. These raw materials are a component in nutraceutical preparations and diet supplements and are used as adaptogenic and anabolic preparations. *R. carthamoides* has also been reported to alleviate physical weakness and mental weariness [6]. Studies have revealed the presence of various types of secondary metabolites such as ecdysteroids, phenolic acids with caffeoylquinic acid derivatives, flavonoids, polyacetylenes, sesquiterpene lactones, and triterpenoid glycosides [6, 7]. Most of the available plant compounds are derived from wild plants or plants cultivated in plantation and involve the destruction of whole plants. Hence, in recent years, researchers have sought potential alternatives in obtaining plant material and valuable compounds with therapeutic effect. One such method is plant biotechnology based on *in vitro* cultures, especially transformed root cultures; these are characterized by high metabolite content and biomass production in a short time. Our previous study established transformed roots of *R. carthamoides* by A4 *Agrobacterium rhizogenes* transformation and showed that these transformed roots contain caffeoylquinic acids and their derivatives and flavonoid glycosides [7]. The major compounds present in these roots are chlorogenic acid, 3,5-*O*-dicaffeoylquinic acid, and a tentatively identified tricaffeoylquinic acid derivative. The transformed roots of *R. carthamoides* also demonstrated enhanced production of tricaffeoylquinic acid derivatives compared to the normal roots of soil-grown plants, and they offer an attractive alternative to conventional cultivation and obtainment of the valuable secondary metabolites.

In reference to our earlier studies regarding the cytotoxicity of *R. carthamoides* transformed root extract against human glioma cells, the aim of the present study is estimate its cytotoxic and genotoxic activities in two human leukemia cell lines: myeloid (K-562) and lymphoid (CCRF-CEM) and lung cancer cell line (A549) by evaluating cell viability, mitochondrial DNA (mtDNA) and nuclear DNA (nDNA) damages, loss of mitochondrial membrane potential, and alteration of mtDNA copy number.

## 2. Materials and Methods

### 2.1. Plant Material

Transformed roots of *R. carthamoides* were previously obtained by the transformation of A4 *Agrobacterium rhizogenes* [7]. The establishment and growth of transformed roots as well as phytochemical analysis (identification and quantification of caffeoylquinic acid derivatives) of transformed roots extract were described in our previous study [7]. The roots of soil-grown plants were used as compared material.

### 2.2. Preparation of Extracts for Biological Study

The lyophilized plant material (10 g dry weight) was extracted with 80% (*v/v*) aqueous methanol as described in our earlier study [8]. The yield (*w/w*) of the aqueous methanol extract (*Rc* TR extract) was 19.07%. The roots of soil-grown plants (*Rc* NR extract) were used as a comparison. The yield of *Rc* NR extract was 18.87%.

### 2.3. Human Cancer Cell Cultures

The following cell lines were used: human lung adenocarcinoma A549 (CCL-185, ATCC) and two human leukemia lines—T lymphoblast CCRF-CEM cells (CCL-119, ATCC) and chronic myelogenous leukemia K-562 (CCL-243, ATCC). The cell lines were obtained from the American Type Culture Collection (ATCC™, Manassas, VA, USA). The A549 cells were cultured in DMEM medium, CCRF-CEM, and K-562 cells in RMPI 1640 medium supplemented with 100 units of potassium penicillin and 100 *μ*g of streptomycin sulfate per 1 mL of culture media and 10% (*v/v*) heat-inactivated fetal bovine serum (FBS). All cell lines were grown in a humidified incubator at 37°C and 5% CO_2_. The cell culture reagents were purchased in Lonza (Basel, Switzerland).

### 2.4. Cell Viability (MTT Assay)

MTT assay was used to determine cell viability after 24-hour treatment with *Rc* TR extract or *Rc* NR extract (0.019-5.0 mg/mL). In brief, A549 cells (1 × 10^4^ cells/well), CCRF-CEM cells (1 × 10^5^ cells/well), and K-562 (1 × 10^5^ cells/well) were seeded in a 96-well plate and cultured overnight in the incubator at 37°C and 5% CO_2_. The medium was then removed and replaced with the fresh medium supplemented with *Rc* TR extract or *Rc* NR extract. The cells were incubated for 24 hours, washed once, and centrifuged (300 × *g* for five minutes at 22°C) and incubated with 0.5 mg/mL of 3-(4,5-dimethylthiazol-2-yl)-2,5-diphenyl tetrazolium bromide (MTT) at 37°C. After four hours, the MTT solution was discarded carefully, and the formazan crystals were dissolved in DMSO. Finally, the absorbance was measured for each well at a wavelength of 570 nm with background subtraction at 630 nm using a BioTek Synergy HT Microplate Reader (BioTek Instruments, Winooski, VT, USA). The relative cell viability was expressed as a percentage relative to the untreated (control) cells defined as 100%. All experiments were performed in triplicate.

### 2.5. Mitochondrial Membrane Potential

The fluorescent dye JC-1 (5′,6,6′-tetrachloro-1,1′,3,3′-tetraethylbenzimidazolylcarbocyanine iodide) was used to estimate mitochondrial membrane potential. The cells were seeded into 96-well black plates with transparent bottom (Greiner) at a density of 1 × 10^4^ cells/well (A549) and 1 × 10^5^ cells/well (CCRF-CEM and K-562) in 50 *μ*L culture medium and allowed to adhere overnight. Next, the cells were incubated for 24 hours with *Rc* TR extract, *Rc* NR extract (IC_50_ concentration), or without *Rc* extract (control cells). Then, the cells were preincubated for 30 minutes with 5 *μ*M JC-1 in the HBSS at 37°C in a CO_2_ (5%) incubator. Finally, the cells were centrifuged (300 × g for 10 min at 22°C) and washed twice with the HBSS. The fluorescence was measured on a BioTek Synergy HT Microplate Reader (BioTek Instruments, Winooski, VT, USA) with the filter pairs of 530 nm/590 nm and 485 nm/538 nm. The results are shown as a ratio of fluorescence measured at 530 nm/590 nm to that measured at 485 nm/538 nm (aggregates to monomer fluorescence). The experiments were repeated three times for each cell line.

### 2.6. Genomic DNA Isolation from the Cell Cultures

The cell samples were incubated for 24 hours with *Rc* TR extract, *Rc* NR extract (IC_50_ concentration), or without *Rc* extract (control cells). Total genomic DNA (nuclear or mitochondrial) was isolated by the QIAamp DNA Mini Kit (Qiagen, Valencia, CA, USA) from 2 × 10^6^ cells, according to the manufacturer's instructions. DNA concentrations were determined by the spectrophotometric measurement of absorbance at 260 nm, and the purities were calculated by A260/A280 ratio using a BioTek Synergy HT Microplate Reader (BioTek Instruments, Winooski, VT, USA). The purified DNA was stored at −20°C until further analysis.

### 2.7. Determination of mtDNA and nDNA Damages—Semi-Long-Run qRT-PCR (SLR-qRT-PCR)

The semi-long-run quantitative RT-PCR (SLR-qRT-PCR) was used to quantify mitochondrial DNA (mtDNA) and nuclear DNA (nDNA) damage [9]. Long and small DNA fragments located in the same mitochondrial or nuclear genomic region were used to determine the degree of mitochondrial DNA (mtDNA) and nuclear DNA (nDNA) damage in the examined regions: *ND1* (mitochondrially encoded NADH:ubiquinone oxidoreductase core subunit 1), *ND5* (mitochondrially encoded NADH:ubiquinone oxidoreductase core subunit 5), *TP53* (tumor protein p53), and *HPRT1* (hypoxanthine phosphoribosyltransferase 1). The primer sequences, SLR-qRT-PCR amplification, and damage calculation were performed according to Bijak et al. [10]. SLR-qRT-PCR amplification was performed on a CFX96™ real-time system (Bio-Rad). All experiments were performed in triplicate.

### 2.8. Mitochondrial DNA Copy Number

Quantitative real-time PCR (qRT-PCR) was used to assess the relative number of copies of human mitochondrial DNA (mtDNA) using nuclear DNA (nDNA) content as a standard. Two genes, *ND1* and *ND5*, were chosen as mitochondrial target and *SLCO2B1* and *SERPINA1* genes as nuclear target. The primer sequences, qRT-PCR amplification, and mtDNA copy number calculation were performed according to the study described by Bijak et al. [10]. Data was collected using the CFX-96 detection system (Bio-Rad, Hercules, CA, USA). Each reaction was performed in triplicate, and negative controls (without template DNA) were also included in each run.

### 2.9. Statistical Analysis

The results are calculated as means ± SD. The normality of the data was determined by the Shapiro-Wilk test, and Levene's test was used to assess the equality of variance. The Kruskal-Wallis test and the one-way analysis of variance (ANOVA) with Tukey's post hoc test were used to determine the significant differences (*p* < 0.05) between the samples (STATISTICA 12.0 software, StatSoft, Poland).

## 3. Results

### 3.1. Cytotoxic Activity of *Rc* Extracts against the Two Human Leukemia Cell Lines (K-562 and CCRF-CEM) and Lung Cancer Cell Line (A549)

**Figure 1:**
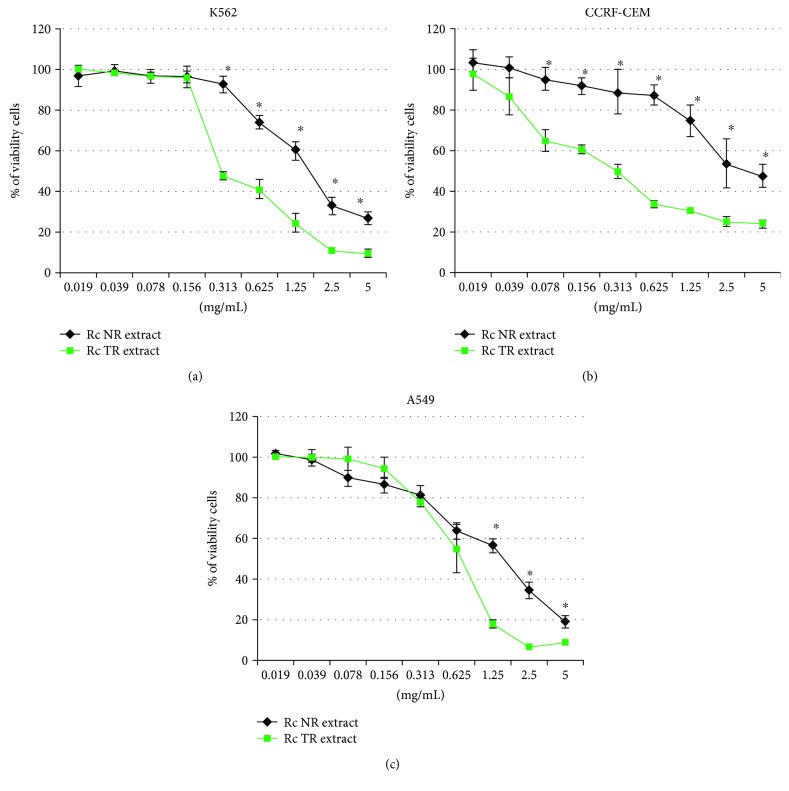
The viability of cell lines K-562, CCRF-CEM, and A549 after 24 h treatment with *R. carthamoides* extracts from the roots of soil-grown plants (*Rc* NR extract) and transformed roots (*Rc* TR extract). The results represent mean ± SD of three independent experiments. ^∗^*p* < 0.05*Rc* NR extract vs. *Rc* TR extract.

Three human cancer cell lines were used in this study: K-562 chronic myelogenous leukemia, CCRF-CEM leukemic lymphoblasts, and A549 lung adenocarcinoma. The cells were treated with different concentrations (0.019-5 mg/mL) of extract from transformed roots of *R. carthamoides* (*Rc* TR extract) for 24 hours. Extract from the roots of soil-grown plants (*Rc* NT extract) was used for comparison. It was found that both *Rc* extracts inhibited the cell viability of all tested cell lines in a concentration-dependent manner (Figure 1), but the *Rc* TR extract possessed stronger properties. Additionally, the two leukemia cell lines (CCRF-CEM and K-562) were more sensitive to *Rc* TR extract than A549 lung adenocarcinoma. The *Rc* TR extract possessed similar cytotoxic activity for both tested leukemia cell lines (K-562 and CCRF-CEM). For these cell lines, 50% of cell viability was achieved after treatment with *Rc* TR extract at a concentration of 0.313 mg/mL (Figure 1). Twice the concentration of *Rc* TR extract was needed to obtain this effect for A549 human lung cancer cells.

### 3.2. Effect of *Rc* Extracts on Mitochondrial Membrane Potential in Human Leukemia Cell Lines (CCRF-CEM and K-562) and Lung Cancer Cell Line (A549)

**Figure 2:**
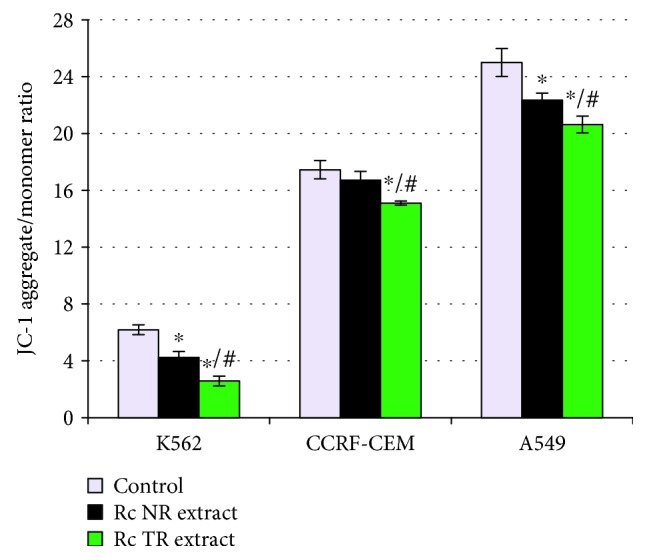
The mitochondrial membrane potential in cell lines K-562, CCRF-CEM, and A549 after 24 h treatment with *R. carthamoides* extracts from the roots of soil-grown plants (*Rc* NR extract) and transformed roots (*Rc* TR extract). To measure mitochondrial membrane potential, the fluorescent dye JC-1 was used. Mitochondrial membrane potential is expressed as ratio of 530 nm/590 nm to 485 nm/538 nm (aggregates to monomer) fluorescence. The results represent mean ± SD of three independent experiments. Control: untreated cells. ^∗^*p* < 0.05 cells treatment with *Rc* NR extract or TR extract vs. control cells; #*p* < 0.05*Rc* NR extract vs. *Rc* TR extract.

The present study also evaluated the permeability of the mitochondrial membrane potential in the two leukemia cell lines and lung cancer cell line treated with *R. carthamoides* root extracts (TR and NR extracts). It was observed that these plant extracts reduced the mitochondrial membrane potential in all tested cell lines compared to the control cells (Figure 2), while the *Rc* TR extract possessed stronger properties. *Rc* TR extract decreased the mitochondrial membrane potential about 2.4-fold in K-562 cells and 1.2-fold in CCRF-CEM and A549 cells.

### 3.3. Quantification of Mitochondrial DNA (mtDNA) and Nuclear DNA (nDNA) Damages in Human Leukemia Cell Lines (CCRF-CEM and K-562) and Lung Cancer Cell Line (A549) after Treatment with *Rc* Extracts

**Figure 3:**
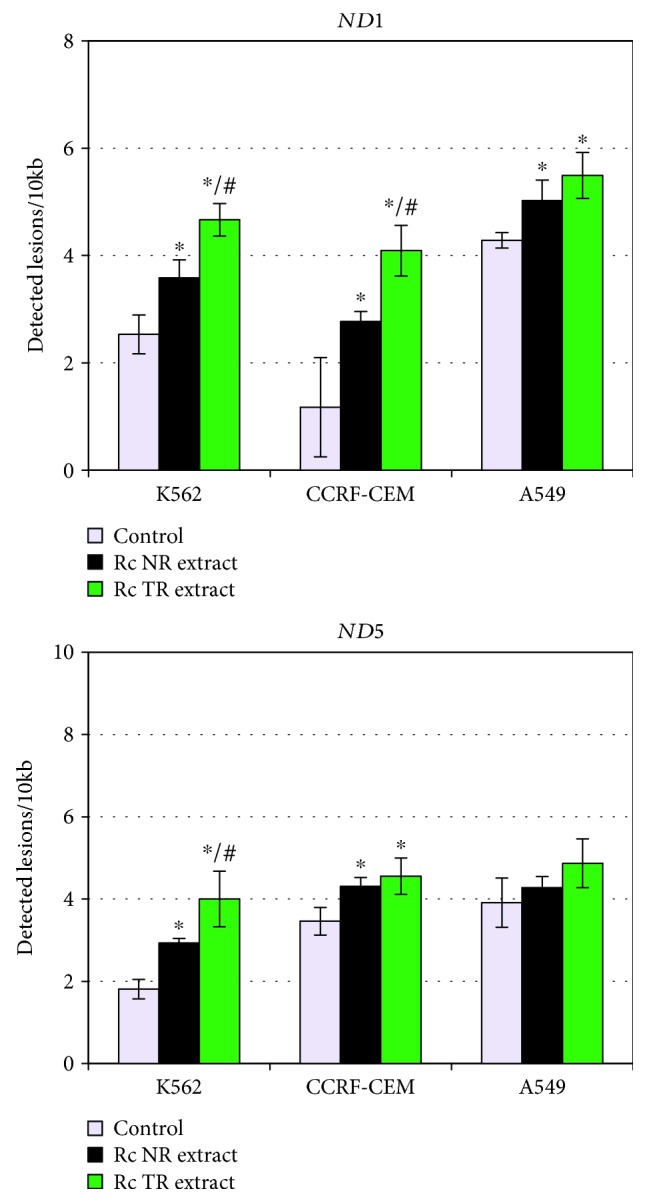
mtDNA damage estimated as lesion frequency per 10 kb in *ND1* and *ND5* genes in cell lines K-562, CCRF-CEM, and A549 after 24 h treatment with *R. carthamoides* extracts from the roots of soil-grown plants (*Rc* NR extract) and transformed roots (*Rc* TR extract). SLR-qRT-PCR was used to quantify mtDNA damage. The results represent mean ± SD of three independent experiments. Control: untreated cells. ^∗^*p* < 0.05 cells treatment with *Rc* NR extract or TR extract vs. control cells; #*p* < 0.05*Rc* NR extract vs. *Rc* TR extract.

The study also examined mtDNA and nDNA damages in three human cancer cell lines after 24-hour treatment with *R. carthamoides* root extracts by SLR-qRT-PCR. The results demonstrated that *Rc* TR and NR extracts possessed genotoxic activity by increasing the level of mtDNA damage in the *ND1* regions in all cancer cell lines and in the *ND5* regions only in human leukemia cell lines (K-562 and CCRF-CEM). The lesion rate in the *ND1* region increased after treatment with *Rc* NR extract and *Rc* TR extract compared to the control cells (cells untreated with roots extracts): 3.6 lesions per 10 kb DNA (*Rc* NR extract) and 4.7 lesions per 10 kb DNA (*Rc* TR extract) in K-562 cells. Furthermore, the *Rc* TR extract also caused a higher lesion rate in the *ND1* region in CCRF-CEM cells compared to the *Rc* NR extract (Figure 3) resulting in 4.1 lesions per 10 kb DNA vs. 2.8 lesions per 10 kb DNA, in turn for A549 cells, 5 lesions per 10 kb DNA in those treated with *Rc* NR extract and 5.5 lesions per 10 kb DNA after *Rc* TR extract (without significant differences). Additionally, an increased level of mtDNA damage was found in the *ND5* region only in human leukemia cell lines: 2.9 lesion per 10 kb DNA (*Rc* NR extract) and 4.0 lesions per 10 kb DNA (*Rc* TR extract) in K-562 cells and 4.3 lesion per 10 kb DNA (*Rc* NR extract) and 4.6 lesions per 10 kb DNA (*Rc* TR extract) in CCRF-CEM cells (Figure 3). No significant differences were found between the tested root extracts for the CCRF-CEM cells.

**Figure 4:**
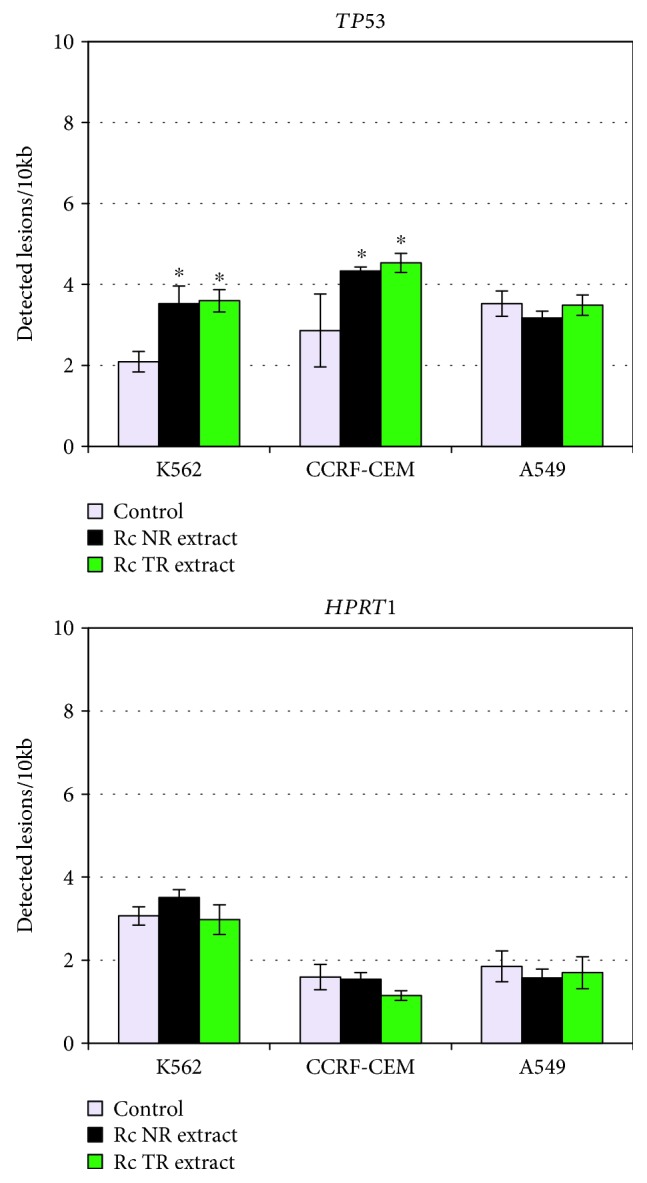
nDNA damage estimated as lesion frequency per 10 kb in *TP53* and *HPRT1* genes in cell lines K-562, CCRF-CEM, and A549 after 24 h treatment with *R. carthamoides* extracts from the roots of soil-grown plants (*Rc* NR extract) and transformed roots (*Rc* TR extract). SLR-qRT-PCR was used to quantify nDNA damage. The results represent mean ± SD of three independent experiments. Control: untreated cells. ^∗^*p* < 0.05 cells treatment with *Rc* NR extract or TR extract vs. control cells; #*p* < 0.05*Rc* NR extract vs. *Rc* TR extract.

The root extracts also exhibited genotoxic activity by increasing nDNA damage in the *TP53* region in the human leukemia cell lines (K-562 and CCRF-CEM), but no significant differences were found between the tested extracts (Figure 4). The results showed that, in K-562 cells, the plant extracts increased nDNA damage by about 3.5 lesions per 10 kb and, in CCRF-CEM cells, about 4.5 lesions per 10 kb. Additionally, neither *Rc* NR nor TR extracts induced damage in the *HPRT1* region in any tested cell line (Figure 4).

### 3.4. Quantification of mtDNA Copy Number in Human Leukemia Cell Lines (CCRF-CEM and K-562) and Lung Cancer Cell Line (A549) after Treatment with *Rc* Extracts

**Figure 5:**
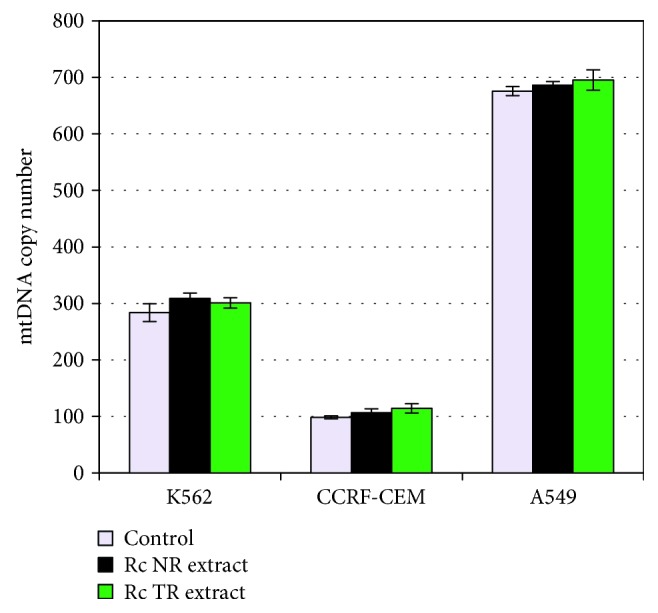
The effect of *R. carthamoides* extracts from the roots of soil-grown plants (*Rc* NR extract) and transformed roots (*Rc* TR extract) on the mtDNA copy number in cell lines K-562, CCRF-CEM, and A549 after 24 h incubation. qRT-PCR was used to assess the mtDNA copy number. The results represent mean ± SD of three independent experiments. Control: untreated cells.

The mtDNA copy number was also determined in three human cancer cell lines: K-562, CCRF-CEM, and A549. It was found that the mtDNA copy number was slightly elevated after *Rc* TR and NR extracts treatment in all tested cancer cell lines, but without significant differences with the control cells (Figure 5).

## 4. Discussion

Cancer, the third leading cause of death worldwide [11], is associated with changes in signaling pathways, which are often induced by mutations in oncogenes or tumor suppressor genes [12].

Our studies from recent years [3–5] have demonstrated that transformed roots extract of *R. carthamoides* possesses cytotoxic activity against human glioma cells and can induce apoptosis by disrupting mitochondrial membrane potential and increasing ROS level, altering Bax/Bcl-2 level, or by activating p53, caspase-3, or caspase-9. This plant extract also demonstrated genotoxic effects against human glioma cells by inducing DNA damage, increasing the number of cleaved PARP1-positive cells, and altering the level of *γ*-H2A.X-positive cells: a marker of double strand breaks in DNA [5]. Hamburger et al. [13] reported that the roots of natural growing plants of *R. carthamoides* suppressed the viability of MCF-7 human breast cancer cells. It is important to note that *Rc* TR extract has not displayed cytotoxicity against normal human astrocytes in a concentration range of 0.1-1.5 mg/mL [3, 4].

It is possible that the caffeoylquinic acid derivatives present in *Rc* TR extract may inhibit human glioma cell viability, by induction of apoptosis, via intrinsic pathway and caspase activation. Many studies have reported that these phenolic compounds, widespread throughout the human diet, possess anticancer activity in various types of cancer cell lines [14–19]. Ekbatan et al. [20] suggest that different polyphenol compounds may demonstrate antiproliferative mechanisms that act by modulating cell-cycle regulators like p53 or by inhibiting molecular pathways based around NF-*κ*B, activator protein 1, or mitogen-activated protein kinase. The *Rc* TR extract in the present study was rich in caffeoylquinic acid derivatives, with the tentatively identified tricaffeoylquinic acid derivative (5.97 mg/g DW), chlorogenic acid (5.12 mg/g DW), and 3,5-*O-*dicaffeoylquinic acid (3.08 mg/g DW) being the main compounds [7].

In order to determine whether the *Rc* TR extract also possesses cytotoxic and genotoxic activity in various types of cancer cell line, three human cancer cell lines were tested: K-562 chronic myelogenous leukemia, CCRF-CEM leukemic lymphoblasts, and A549 lung adenocarcinoma. It was found that extract from *R. carthamoides* transformed root inhibited the cell viability of all tested cell lines in a dose-dependent manner (0.019-5 mg/mL) and that the leukemia cell lines CCRF-CEM and K-562 were more sensitive to *Rc* TR extract than A549 lung adenocarcinoma. For leukemia cell lines, the IC_50_ was 0.313 mg/mL, and for lung cancer cells, IC_50_ = 0.625 mg/mL. Mahbub et al. [21] reported that leukemia cell lines demonstrate varying sensitivity to polyphenol treatment, with CCRF-CEM lymphoid cell line being more sensitive than K562 myeloid cell line. In the present study, both leukemia cell lines showed the same level of sensitivity to *Rc* TR extract. Additionally, our findings indicate that the *Rc* TR extract was more cytotoxic to the human leukemia and lung cancer cell lines than human patient-derived grade II-IV glioma cell lines and U87MG human glioma cells [3–5]. The *Rc* TR extract reduced also the viability of glioma cell lines in a dose-dependent manner, reaching about 50% at a concentration of 0.75 mg/mL or 1.0 mg/mL. The differences observed in cells sensitivity to *Rc* TR extract treatment may reflect the specificity of the tissue or the degree of malignancy.

In chemotherapy, the induction of apoptosis in cancer cells plays an important role [22] and is central to the action of the drug [23]. Mitochondria play important roles in apoptosis induction [24]. An increase of mitochondrial ROS is associated with mitochondrial damage [1, 25] and can induce DNA damages, alkali labile sites, single strand breaks, and damages to purines and pyrimidines [26]; it can cause the dysfunction of mitochondria, protein oxidation, and lipid peroxidation [25]. Cancer cells are characterized by lowered mitochondrial respiration and elevated glycolysis associated with ATP production during glucose metabolism [27]. ROS produced in the cancer cells can activate several transcription factors including activator protein-1, NF-k*β*, and STAT3 which are essential in controlling cellular proliferation, tumor survival, and angiogenesis [26]. The balance of ROS and antioxidant levels plays an important role in apoptosis in cancer cells, and an increase in ROS production could thus promote apoptosis [1] and inhibit cancer cell viability. High ROS level can induce a loss of mitochondrial membrane potential in the cells. Our present findings indicate that *Rc* TR extract reduced the mitochondrial membrane potential in all tested human cancer cell lines: K-562 chronic myelogenous leukemia, CCRF-CEM leukemic lymphoblasts, and A549 lung adenocarcinoma. It has been previously found that *Rc* TR extract can disrupt mitochondrial membrane permeability in human glioma cells and can cause a decrease in mitochondrial membrane potential [3, 4]. Chlorogenic acid, one of the main compounds in *Rc* TR extract, was found earlier to reduce the mitochondrial membrane potential in U937 leukemia cells [28].

In cancer cells, mtDNA mutations can be induced by ROS and reactive aldehydes, resulting in changes in encoded proteins needed for mitochondrial functions [25]. The present study also examines the effect of *Rc* TR extract on mtDNA damage in tested cancer cell lines (K-562, CCRF-CEM, and A549). Our results show an increase level of mtDNA damage in the *ND1* region in all cell lines and in the *ND5* region only in the leukemia cell lines after treatment with *Rc* TR extract compared to control, untreated cells.

As the release of mitochondrial ROS can cause damage of nuclear DNA, leading to the promotion of apoptosis and activation of transcription factor [25], the study also investigates nuclear DNA (nDNA) damage. Our results indicate that *Rc* TR extract increased nDNA damage in the *TP53* region in both leukemia cell lines: K-562 and CCRF-CEM. No difference in the amount of nDNA damage in the *TP53* region was observed between A549 cells treated with *Rc* TR extract and control. Additionally, no significant difference in the level of nDNA damage in the *HPRT1* region was found in all three tested cancer cell lines. Our previous study indicated that 24-hour treatment with *Rc* TR extract resulted in the induction of DNA double strand breaks in human glioma cells [5]. The authors attribute this increased DNA damages and elevated phosphorylated H2A.X level may be due to the cleavage and inactivation of PARP1 and/or the inhibition of its synthesis. Burgos-Morón et al. [29] observed that chlorogenic acid, a major chemical compound in the *Rc* TR extract, induced DNA damage in K-562 leukemia cells.

Currently, much interest is directed to mtDNA copy number for assessing the functioning of mitochondria. As mitochondria might play a key role in cancer susceptibility and development, it is reasonable to speculate that mtDNA variations or changes of mtDNA copy number may be closely related to various cancers [30]. As reported by Gisbergen et al. [31] and Barrera et al. [25], alterations in mtDNA copy number have been observed in different cancer cell lines including glioblastoma, lung carcinomas, or esophageal squamous cell carcinoma; however, their role in cancer is still unknown. In the present study, mtDNA copy number was slightly elevated in all tested cancer cell lines treated with *Rc* TR extract but not significantly. Both increases and decreases in the mtDNA copy number elevate the risk of tumorigenesis [25, 31], and low copy number in cancer cells can be responsible for tumor progression [31] or for chemotherapy resistance [32]. mtDNA copy number reduction has also been found to increase multidrug resistance 1 (MDR1) gene expression, with higher tolerance to anticancer agents in human osteosarcoma 143B cells, colon cancer HCT-8 cells, and hepatoma cells [33]. In turn, Mei et al. [34] suggest that lower mtDNA copy number may cause an increase of ROS level in cancer cells and increase the sensitivity to chemotherapeutic drugs by the induction of apoptosis. One possible approach to cancer therapy may be based on maintaining an adequate balance in mtDNA content during the induction of apoptosis [20]. However, Gisbergen et al. [31] reported both elevated or lowered mtDNA copy number for the same type of cancer, suggesting that mtDNA variation in cancer cells remains unclear and further research is required.

## 5. Conclusion

Both the present and earlier findings demonstrate that transformed roots of *R. carthamoides* can inhibit cell viability and induce apoptosis in various types of cancer cell (such as glioma cells, leukemia cells, or lung adenocarcinoma cells). These transformed roots, rich in caffeoylquinic acid derivatives, may be an effective drug in cancer treatment. Additionally, the present study showed that *Rc* TR extract reduced the mitochondrial membrane potential in all tested human cancer cell lines and possessed genotoxic activity by increasing mtDNA and nDNA damages. *Rc* TR extract shows potent anticancer activity in various cancer cells by inducing mitochondrial dysfunction, but mechanism of action is not clear yet and it needs further studies.

## Data Availability

All data used to support the findings of this study are available from the corresponding author upon request.
